# Optimization of sparse phenotyping strategy in multi-environmental trials in maize

**DOI:** 10.1007/s00122-025-04825-y

**Published:** 2025-02-28

**Authors:** S. R. Mothukuri, Y. Beyene, M. Gültas, J. Burgueño, S. Griebel

**Affiliations:** 1 Faculty of Agriculture, University of Göttingen, Büsgenweg 5, 37077 Göttingen, Germany; 2 Global Maize Program, International Maize and Wheat Improvement Center, ICRAF House, United Nations Avenue, 41Village Market Gigiri, Nairobi, 00621 Kenya; 3 Faculty of Agriculture, South Westphalia University of Applied Sciences, Lübecker Ring 2, 59494 Soest, Germany; 4 Biometrics and Statistics Unit, International Maize and Wheat Improvement Center, Carretera México-Veracruz, Km. 45, El Batán, 56237 Texcoco, México; 5 Department of Crop Sciences, Faculty of Agricultural Sciences, Georg-August-University Göttingen, 37075 Göttingen, Germany

## Abstract

**Key message:**

The relatedness between the genotypes of the training and the testing set using sparse phenotyping experiments helps optimize the line allocation by utilizing the relationship measurements to reduce cost without compromising the genetic gain.

**Abstract:**

The phenotyping needs to be optimized and aims to achieve desired precision at low costs because selection decisions are mainly based on multi-environmental trials. Optimization of sparse phenotyping is possible in plant breeding by applying relationship measurements and genomic prediction. Our research utilized genomic data and relationship measurements between the training (full testing genotypes) and testing sets (sparse testing genotypes) to optimize the allocation of genotypes to subsets in sparse testing. Different sparse phenotyping designs were mimicked based on the percentage (%) of lines in the full set, the number of partially tested lines, the number of tested environments, and balanced and unbalanced methods for allocating the lines among the environments. The eight relationship measurements were utilized to calculate the relatedness between full and sparse set genotypes. The results demonstrate that balanced and allocating 50% of lines to the full set designs have shown a higher Pearson correlation in terms of accuracy measurements than assigning the 30% of lines to the full set and balanced sparse methods. By reducing untested environments per sparse set, results enhance the accuracy of measurements. The relationship measurements exhibit a low significant Pearson correlation ranging from 0.20 to 0.31 using the accuracy measurements in sparse phenotyping experiments. The positive Pearson correlation shows that the maximization of the accuracy measurements can be helpful to the optimization of the line allocation on sparse phenotyping designs.

**Supplementary Information:**

The online version contains supplementary material available at 10.1007/s00122-025-04825-y.

## Introduction

Selection decisions in plant breeding are based on information extracted from phenotypic data generated in multi-environmental trials (METs). Even when new technologies such as genotyping and high-throughput phenotyping provide cheaper genotype data, phenotyping continues to be a critical step in plant breeding. Crossa et al. (2017) affirm that phenotypic evaluation is the most significant bottleneck currently affecting plant breeding initiatives. Combining phenotypic, genotypic, and high-throughput data helps breeders improve breeding value estimation and reduce the cost of plant breeding programs. It is crucial to optimize the allocation of resources to different stages of the breeding pipeline with limited resources. Developing effective genomic-enabled prediction models, including genotypic by environmental (GEI) and other covariates, could optimize resource allocation and increase yield gain without considerably raising costs (Jarquín et al. 2017). Predictive models can be utilized in the context of METs to reduce phenotypic costs with the amount price per precision. This loss in precision could be recovered by increasing the population size, increasing the sampling of the target population environments, and the length of the breeding cycle. Sparse phenotyping consisting of testing some genotypes in the field and predicting partially phenotyped and genotyped is a methodology gaining adeptly shown value in plant breeding. However, as with any new method, many questions remain open. In this study, we are interested in optimizing the sparse phenotyping design by deciding how to allocate lines, e.g., which genotypes will be tested in all environments, and which ones will be evaluated in some environments (Sparse evaluation).

Improvements in DNA sequencing have provided high-throughput, quick, and relatively cheaper genotypic information, improving genomic prediction (GP) in plant and animal breeding, Meuwissen et al. (2001). The procedures continue to present a new genotyping platform: repeat amplification sequencing (rAmpSeq), which scores hundreds of markers for less than $5 per sample (Buckler et al. 2016). In GP, a training population fits a predictive model for a set of candidate genotypes. The untested genotypes can be predicted using data from other environments and relatives (Burgueño et al. 2012). Predictive models can be utilized in METs to reduce the experimental effort by employing sparse testing techniques in which some genotypes are evaluated in all environments while the remaining genotypes are assessed in some environments. The challenge is to design an economically feasible METs system that conserves land and other resources without compromising breeding genotype performance measurement, prediction, and selection. Different types of sparse phenotyping methodologies simulating prospective applications have been documented in the environment of structured data consisting of year cohorts with phenotypes recorded in various environments (Atanda et al. 2021a, b; Burgueño et al. 2012; Jarquin et al. 2020). Sparse testing lowers the cost of field evaluation at a fixed capacity or increases the overall evaluation capacity at a fixed cost, resulting in an increase in selection accuracy through better coverage of the evaluated environments and potentially improving selection gains (Crossa et al. 2017; Isidro y Sánchez and Akdemir 2021; Jarquin et al. 2020).

An essential factor when planning sparse testing is the statistical models to compute the breeding value of the genotypes during our research. Genome-enabled prediction models have been used to improve selection accuracy by incorporating predictions as additional phenotypes (Guo et al. 2020; Jarquin et al. 2020; Jarquín et al. 2014) to reduce the duration of the cycle by omitting some stages (Crossa et al. 2017) and to reduce experimental effort by testing only subsets of the considered genotypes (Burgueño et al. 2012), thereby increasing evaluation capacity and potentially selection intensity. The first proposed whole-genome regression approaches are based on the availability of thousands of genome-wide molecular markers to estimate genomic breeding values (Meuwissen et al. 2001) in plants (Crossa et al. 2010; de los Campos et al. 2009; Pérez et al. 2010). In general, and based on the previous, Crossa et al. (2017) have shown that genomic-enabled models, including GEI, improve prediction accuracy models incorporating genomic information with genomic similarities offer the best prediction accuracy (Burgueño et al. 2012; Cuevas et al. 2016, 2017; Jarquín et al. 2014, 2017; Lopez-Cruz et al. 2015).

Modeling the GEI by a parsimonious statistical model to capture the unstructured phenotypic variance–covariance matrix, such as the factor analytic (FA) model, significantly increased maize prediction accuracy when incorporating the genomic relationship matrix. Since the FA is a linear mixed model, it has advantages to error variance modeling can be accommodated (Burgueño et al. 2012; Crespo-Herrera et al. 2017, 2018). Recent studies have shown applying GEI-based GP models for quantitative traits (Burgueño et al. 2012; Heslot et al. 2014; Jarquín et al. 2014). Burgueño et al. (2011) found that FA models have analyzed better at prediction accuracy of the outcome by 6% than models with the same variance and correlation across environments. Lopez-Cruz et al. (2015) have presented that GP models considering GEI produced more accurate predictions.

Studies concentrating on optimizing sparse testing procedures to maximize genetic gain are critical in plant breeding. Optimizing training and methods aim to select the minimum number of most informative genotypes for model training (Isidro y Sánchez and Akdemir 2021; Kadam et al. 2021). Recent studies have demonstrated that prediction accuracy decreases when training populations are unrelated to the testing population in plant and animal breeding (Albrecht et al. 2014; Lorenz and Smith 2015; Windhausen et al. 2012). In plant breeding, prediction accuracy levels vary greatly depending on the size of the training population, the relationship between the training and testing sets, trait complexities, marker density, and genotyping platforms (Bian and Holland 2017; Zhao et al. 2012). Norman et al. (2018) have shown that enhancing genetic variation within the training set could improve prediction accuracy, mainly when the relatedness between the training and testing sets is minimal.

There are many strategies for optimizing the training set, two of which are to minimize the prediction error variance (PEV) of the resulting predictions on the target population and maximize the coefficient of determination (CD) of the predictions (Akdemir 2017; Guo et al. 2020; Isidro y Sánchez and Akdemir 2021; Lorenz and Nice 2017). Optimizing the training set includes selecting a subset of training individuals that accurately predict not phenotyped germplasm in a testing set (Isidro y Sánchez and Akdemir 2021). Akdemir (2017) research has shown different methods to optimize a training set to select the best-performing individuals in the training set. The selection of training populations by genetic algorithm (STPGA) calculates the relationship between training and testing sets to optimize line allocation in METs, showing higher prediction accuracy than alternative methods (Akdemir 2017). Isidro y Sánchez and Akdemir (2021), Lorenz and Smith (2015), and Rincent et al. (2017) addressed the genetic relatedness between training and testing sets. Recently, Atanda et al. (2021a, b) utilized the average genetic association between specific genotypes in the training and genotypes in the testing set. They found a statistically significant increase in the accuracy compared to the CD in various maize biparental populations.

Increased testing environments throughout these stages of GP may significantly improve selection accuracy compared to the advanced stages, where the majority of selection candidates are actively phenotyped in different environments (He et al. 2016). Because there are many resources and there is an issue with how to use them to get the most genetic gain at the lowest possible cost. Even though it is typically impossible to evaluate all genotypes in all environments due to limited resources, observing some of these genotypes enables us to examine the marker alleles in all environments and the GEI in abundance. Information about the Marker’s response patterns and how they interact with their surroundings could significantly enhance the ability to predict lines that have not been tested in the environment. In this way, sparse testing is essential for making METs cost-effective, but it does not significantly affect the accuracy of the field trials. Breeders can face practical issues without having marker data because of the not-tested genotypes using sparse phenotyping experiments. Breeders can face the issue of having insufficient phenotyping data. Using real sparse phenotyping, breeders can face problems by not obtaining marker data to perform genomic best linear unbiased predictor (GBLUP); it is an issue for the breeders to make predictions based on the genomic information to improve prediction accuracy. Breeders do not have the complete data for particular environments because of not growing or not germinating genotypes from the tested METs, etc.

Our main objective is to evaluate alternatives to allocate lines to sets using different genetic relationship measurements (RMs) between the lines tested in all environments and sparse tested lines. Our research is conducted by using METs from the International Maize and Wheat Improvement Center (CIMMYT) global maize program (GMP) in Africa to mimic different sparse designs to improve the genetic gain without compromising the precision by reducing phenotypic cost.

## Materials and methods

### Datasets

The test crosses of 304 double haploid lines crossed by three testers were evaluated under field conditions in Kenya in 2018. The plant material tested originates from intermediate maize varieties with drought tolerance, nitrogen use efficiency, and resistance to major diseases from CIMMYTs global maize program in Africa. It is adapted to Eastern African tropical rainfed mid-altitude (1000–1800 masl) agro-ecologies that are dry (600–800 mm annual rainfall) or wet (900–1500 mm/year).

The 912 test crosses were evaluated in five environments in Kenya: Embu, Kaguru, Kakamega, Kiboko, and Kirinyaga, under optimal management conditions in the 2018 season. The testers were single cross hybrids of CIMMYT lines, namely, CML322/CML543, CML395/CML444, and CML543/CML566. For practical reasons, the 912 test crosses were divided into seven sets per environment (Suppl. Table 1). Each set was tested in an alpha (0, 1, 2) design, meaning seven field experiments were planted in each environment. Four to six commercial checks were tested with the test crosses in each environment. Each set was organized into blocks and varied from 20 to 47 blocks. The number of test crosses per experiment ranged from 78 to 183 (Suppl. Table 1).

**Fig. 1:**
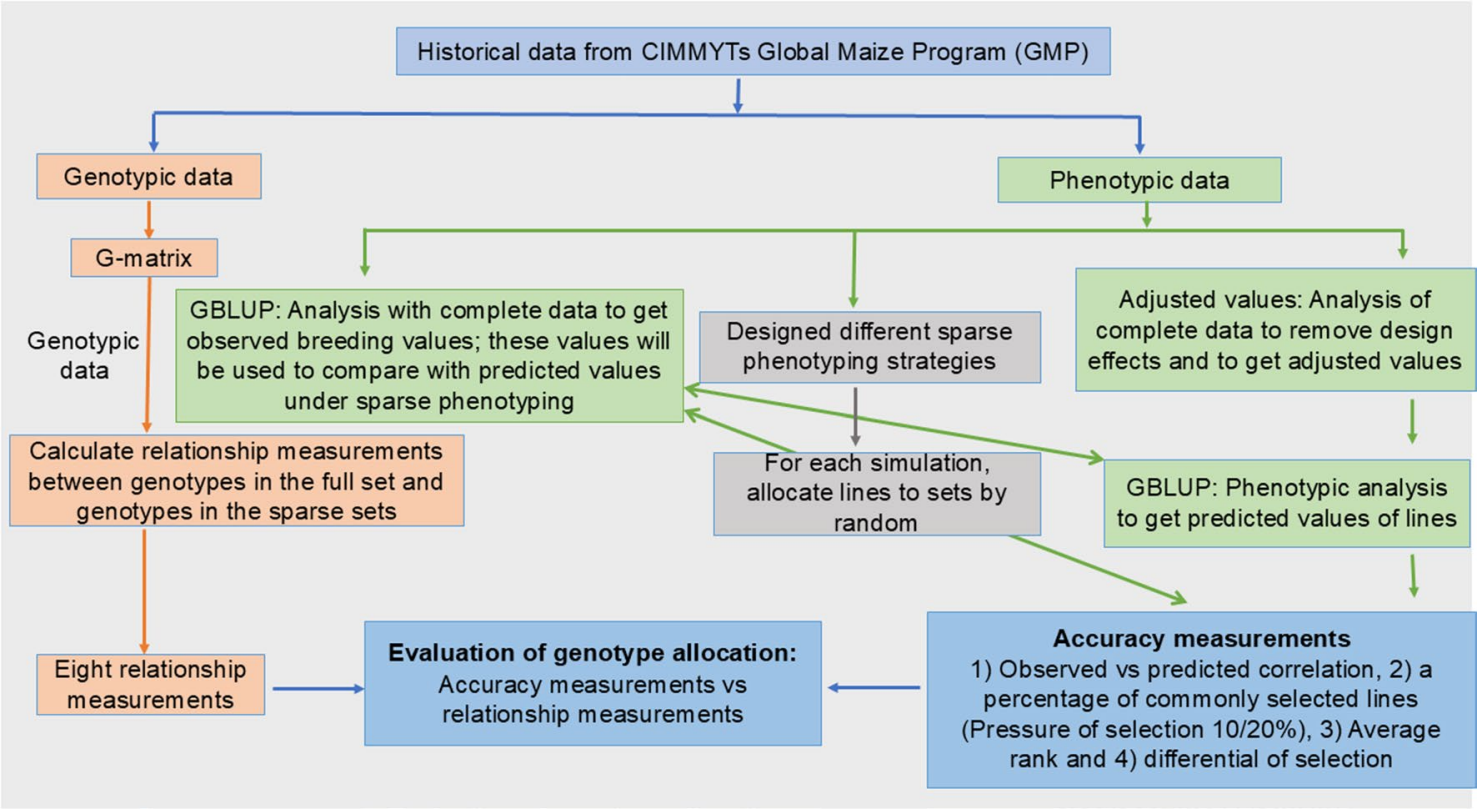
Illustrates the stepwise approach to optimize the line allocation in sparse phenotyping designs using GP and RMs in CIMMYT’s tropical maize breeding program

Four phenotypic traits were collected, namely, grain yield (GY), ears per plant (EPP), plant height (PH), and ear height (EH). The fresh ear weight and the 12.5% of the adjusted moisture content were used to calculate the GY in t/ha. The EPP calculated the number of ears and the number of plants counted, and then, the number of ears was divided by the number of plants. The PH was measured at the flowering time as the centimeters distance between the plant’s base to the bottom of the flag leaf. The EH of the test crosses per plot was measured meters from the ground level to the base of the uppermost cobs bearing internode. Figure 1 illustrates the stepwise approach to optimize line allocation in sparse phenotyping designs using phenotypic and genotypic data.

### Genotypic data

The 304 DH lines were sequenced with rAmpSeq’s low-cost per sample genotyping platform, Ed Buckler Lab at Cornell Life Science Core Laboratory, Ithaca, NY, USA (Buckler et al. 2016). In total, 3532 dominant markers out of 4489 markers are used in this study. The subset of dominant markers has allele frequencies between 0.05 and 0.95. The 21.31% of the markers have allele frequencies less than 0.05, which have been removed to calculate the relationship matrix. Figure 1 illustrates the stepwise approach to optimize line allocation in sparse phenotyping designs using phenotypic and genotypic data.

### Sparse designs

To define a sparse design, some parameters must be determined in given a number of environments and a number of genotypes, genotypes must be classified into different sets, and each set must be tested in a given environment. To better estimate the GEI, a genotype set is tested in all environments while the others are phenotyped in a few environments. Because all test crosses were tested in all environments, we can mimic different sparse designs and evaluate which provides better results in terms of cost and precision.

The not-tested environments for sparse sets (SSs) are based on the number of possible combinations to design balanced strategies, while to design unbalanced sparse strategies, not-tested sparse sets are decided without considering possible combinations. Here, information from relatives is utilized, and the prediction evaluation can gain from borrowing information between lines inside an environment, between lines across environments, and among associated environments. Sparse testing is similar to incomplete field trials. The incomplete field trials are designed by using a certain portion of tested genotypes in a specific portion of tested environments. Sparse phenotyping uses related lines and correlated environments, and prediction assessment gains from borrowing information across lines within an environment, between lines in different environments and between correlated environments (Burgueño et al. 2012).

Different sparse phenotyping schemes were designed based on the proportion of lines assigned to the FS, the number of tested environments for SSs, and balanced and unbalanced sparse designs. Our research included 30% (104 unique lines) and 50% (154 unique lines) lines into the FS in the SP1–SP7 and SP8–SP14 strategies. The different % of lines were assigned to the FS to find which line proposition was helpful in terms of precision in order to optimize the line allocation. The number of tested environments in each SS differentiates the methods in terms of the cost, precision, and performance of the RMs in sparse phenotyping designs.

**Fig. 2:**
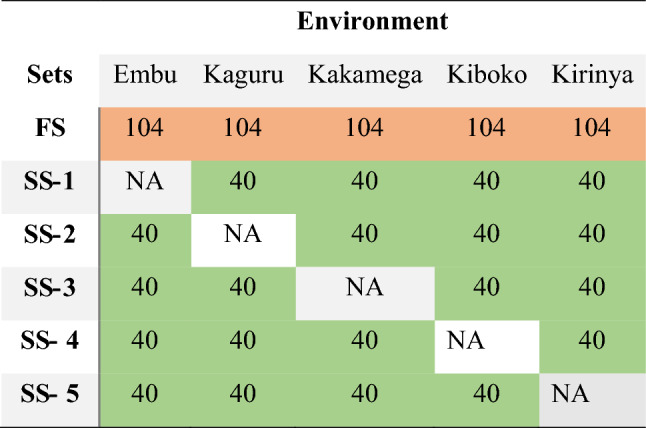
One example of a sparse phenotyping strategy was used above the figure to illustrate the allocation of lines into sets to mimic several sparse designs. The distribution of 304 lines was split into six groups for each environment. The figure’s red cells have been analyzed in all conditions. The green cells in sparse set-1 (SS-1) through SS 5 were the SSs that were not examined in a single environment but were evaluated in four environments. The uncolored cells represent the not-contained tested sets in each environment

Due to budget constraints, the number of genotypes that can be tested in the field is limited. We cannot phenotype 1824 test crosses (304 lines * 3 testers * 2 rep) to each environment in the field because of the limited financial resources. The 304 unique lines were randomly split into FS and SSs. The FS was evaluated in five environments, while SSs were tested in four environments. Approximately 30% (104 unique lines) of the lines have been assigned to the FS and tested in all environments. Around 70% (200 unique lines) of the lines were randomly allocated into the SSs and equally split into five unique SSs. Each SS had 40 lines and was assessed in four different environments (Fig. 2). For each design, 100 random assignations of lines to sets were performed to obtain the 100 simulations in order to produce the 100 cross-validation repetitions. The lines were allocated to the sets according to Table 1 in order to run the 100 repetitive prediction models. A total of 1400 simulation data were obtained to mimic the 14 sparse phenotyping designs for each trait (14 designs * 100 simulations * 4 traits = 5600 simulations) to predict the not-tested lines using the GBLUP and calculate the accuracy measurements (AMs).

**Table 1:** This study used 14 sparse phenotypic designs

Number of SSs (+ one FS)	Number of tested environments for SS	30% (104) of the lines allocated to the FS designs	% of the plots saved by using SP1–SP7	50% (154) of the lines assigned to the FS	% of the plots saved by utilizing the SP8–SP14
5	4	SP1	13.15	SP8	9.86
10	3	SP2	26.31	SP9	19.74
10	2	SP3	39.47	SP10	29.61
5	1	SP4	52.63	SP11	39.47
1	0	SP5	65.78	SP12	49.35
5	3	SP6	26.31	SP13	19.74
5	2	SP7	39.47	SP14	29.61

The first column displays how many SSs were used in each environment to simulate each design. The second column illustrates the number of environments tested for each sparse set (SS). The 4th and 6th columns represented the % of the saved plots. Column 3 shows the sparse phenotyping 1 (SP1)

Designs SP1–SP7 included 30% (104 unique lines) of the lines to the full set (FS), but SP8–SP14 contained 50% (154 unique lines)

### Relationship measurements (RMs) on sparse phenotyping strategies

RMs were utilized to calculate the relatives and the strength of their relationship between FS and SS genotypes on the 14 sparse phenotyping designs. Based on 14 sparse phenotyping methods, RMs were used to identify measurements helpful for optimizing line allocation to minimize the phenotyping resource and enhance the genetic gain.

The STPGA package includes instructions for optimizing the training set and calculating the relationship values between the training and testing sets by utilizing various RMs (Formulas explained in the STPGA package) (Akdemir 2017). The STPGA has been used to calculate the relationship between the FS and SS for each sparse design to determine which relationship measurement provides a better relationship and provides significant correlations to optimize the line allocation.

In this study, eight RMs were used to evaluate the genomic relationship between FS and SS. Prediction error variance mean (PEVMEAN) was calculated as the mean of the diagonal elements of the prediction error variance matrix, reflecting the average prediction accuracy, while prediction error variance maximum (PEVMAX) identified the maximum prediction error variance based on the largest diagonal value. Negative distance in training (neg_dist_in_train) measured the genetic distance in the FS as the mean distance from the FS to the test set using a distance matrix. Genomic optimal prediction–prediction error variance (GOPTPEV) was computed as the maximum eigenvalue of the genomic prediction matrix, optimized by minimizing the prediction error variance to enhance prediction reliability. Distance to optimal prediction (DOPT) quantifies the distance to optimal prediction using the log determinant of the prediction error variance matrix. The coefficient of determination mean (CDMEAN) is the average coefficient of determination, indicating the overall genetic similarity between FS and SS, while the coefficient of determination max (CDMAX) captures the maximum coefficient of determination, identifying the strongest genetic relationships. Lastly, the accuracy of the optimal prediction (AOPT) was calculated using the trace of the inverse of the genomic prediction matrix, providing a measure of prediction accuracy within the sparse phenotyping design.

The genetic relationship matrix was derived from the array of markers (n × m) (n: number of genotypes), (m: number of markers) coded as 0, 1. The principal components of the genetic relationship matrix were computed, and 304 components were selected for error variance estimation. To determine the relationship values for the “dist to test” measurement, models required the input of the distance matrix computed from marker data. The distance matrix was calculated with the STPGA-R package. This study has not performed the optimization step on the FS; instead compared and assessed the resemblance between the FS and SS and statistical values using a random sample of genotypes from the population structure study (Windhausen et al. 2012).

STPGA has R-instructions to calculate the RMs between the FS and SSs of the genotypes.

1 $$CDMAX \, \left( {FS, \, SS, \, P, \, lambda \, = \, 1e - 05} \right)$$

2 $$dist\_to\_test \, \left( {FS, \, SS, \, Dst, \, lambda \, = \, 1e - 05} \right)$$

The FS vector included unique identifiers for each individual of the FS; the SS vector contained unique identifiers for each individual of the SS. *P* is an n×k matrix of the predictor variable's initial principal component analysis (PCA). As row names, the matrix must contain similar to the full and sparse individual’s identities; lambda is a scalar shrinkage parameter (*λ* > 0), and Dst is an n×n symmetric distance matrix with row and column names.

### Study design to define an allocation method

The four AMs and RMs were used to evaluate the different sparse designs. The AMs were Pearson correlation, the common % of the selected lines, average rank, and differential of selection. The Pearson correlation was utilized to calculate the correlation between the observed breeding value (OBV) and predicted breeding value (PBV) from the 304 lines to evaluate the performance of the sparse designs in terms of accuracy with the % of saved plots. The common % of the selected lines, average rank, and differential of selection must be calculated from the top performing selected 10% lines (Top 30 lines from the 304 lines). The 10% (30 unique lines) lines were selected from the FS and SS per the design. The % of commonly selected lines was determined by the proportion of the lines selected habitually from the OBV and PBV. The average rank was calculated using OBV and the rank of the OBV on the selected lines in the PBV. The selection differential was computed by subtracting the mean of selected lines from the mean of all lines. The Pearson correlation coefficient has been utilized to determine the significant Pearson correlation between the AMs and RMs to estimate the performance of the relationship and AMs on the sparse phenotyping design in order to optimize the line allocation.

### Statistical analysis

#### Describe the analysis

A mixed model was built to extract the fixed and random effect coefficients. The adjusted response values were calculated by removing the four different effects. The genotypic and phenotypic variance have been used to quantify broad-sense heritability. The genomic inverse matrix (G-inverse matrix) was computed by using the molecular marker data. The genomic-enabled prediction model used the G-inverse matrix and FA model to predict the not-tested test cross in each sparse phenotyping design.

#### Model 1: adjusting response values

A linear mixed model was built using ASREML-R (Butler et al. 2017) to estimate the variance component and assess the main effects for traits such as GY, EH, PH, and EPP across the METs. The following linear mixed model Eq. (1) was used to measure the coefficients.

3 $$Y_{ijklmn} = \mu + \, S_{n} + \, E_{m} + \, SE_{mn} + \, R\left( {SE} \right)_{inm} + \, B\left( {SER} \right)_{ijmn} + L\left( E \right)_{km} + \, T_{l} + \, LT\left( E \right)_{klm} + \, SL\left( E \right)_{knm} + \, ST_{\ln } + \, SLT\left( E \right)_{klmn} + \, e_{ijklmn}$$

where the plot observation takes place, and Y_ijklmn_ was modeled by computed using the best linear unbiased estimators (BLUEs) model. The fixed effects; µ was observed values; S was the effect of the n^th^ environments; E_m_ was the effect of the m^th^ experiments; SE_mn_ was the effect of the interaction of the n^th^ environments m^th^ experiments; and R(SE)_inm_ was the effect of i^th^ replicants in the n^th^ environments in the m^th^ experiments. The random effects; B(SER)_ijmn_ was the effect of the j^th^ blocks in n^th^ environments in m^th^ experiments in the R^th^ replicants; L(E)_km_ is the effect of the k^th^ lines nested by m^th^ experiments; T_l_ was the effect of the l^th^ testers; LT(E)_klm_ was an interaction effect between the k^th^ lines and l^th^ testers nested with m^th^ experiments; SL(E)_knm_ is the effect of the interaction of the n^th^ environments in k^th^ lines nested with m^th^ experiments; ST_ln_ was an interaction effect of the n^th^ environments in l^th^ testers; SLT(E)_klmn_ is an interaction effect of n^th^ environments in k^th^ lines, l^th^ testers nested with m^th^ experiments; and e_ijklmn_ was error variance.

Above, the model (Eq. 1) was implemented to estimate the variance components and calculate the coefficients. The fixed and random effects were estimated to calculate the adjusted values for every possible effect. Adjusted values were measured by removing the experimental, environmental, replicant, environmental by experimental, and block effects in Excel. Complete data analysis to eliminate design effects and produce adjusted values to mimic sparse designs.

The broad sense of heritability (H^2^) from Falconer and Mackay (1996) was determined using the variance components obtained by refining the models with all as random effects using the linear mixed model (Eq. 1).

4 $$H^{2} = \frac{{V_{L} }}{{V_{P} }}$$

V_L_ is the line additive variance; and V_P_ is the phenotypic variance and was deliberated as the variance between the lines.

The formula for phenotypic variance (V_P_) follows:

5 $$V_{P} = V_{L} + \frac{{V_{L \times E} }}{{m}} + \frac{{V_{LT} }}{{t}} + \frac{{V_{LTE} }}{{tm}} + \frac{{V_{R} }}{{tmr}}$$

The phenotypic variance (V_P_) is calculated using the following components: V_L_, which is the line variance; ${\text{V}}{\text{LxE}}$, the variance due to the interaction between lines and environments; ${\text{V}}{\text{LT}}$, the variance due to the interaction between lines and testers; ${\text{V}}{\text{LTE}}$, the variance due to the interaction among lines, testers, and environments; and ${\text{V}}{\text{R}}$, the residual variance, which accounts for error variance. The total phenotypic variance is partitioned by considering the number of environments (m), testers (t), and replicates (r) within the study design.

#### Genetic inverse matrix

The genetic relationship matrix was calculated between individuals following the study of VanRaden (2008). The total number of markers was coded as 0 and 1, which demonstrated the absence and presence of the marker, respectively (Buckler et al. 2016).

6 $$G_{VR} = \frac{{ZZ^{\prime } }}{{2\sum {p_{j} \left( {1 - pj} \right)} }}$$

The elements of matrix Z are z_ij_, where z_ij_ represents the genotype as the number of copies of the major frequency allele of DH line i at marker j, marked by 0 or 1 for recessive and dominant homozygotes, respectively, and p_j_ represents the allele frequency at marker j. Due to the fact that the lines were DH, it was presumed that all lines containing the major frequencies were homozygous dominant. The denominator is calculated such that the numerator relationship matrix is the expectation of the genome relationship matrix. AGHmatrix has been utilized to calculate the genomic relationship matrix (Amadeu et al. 2016). The G matrix was ill-conditioned because two full-sib genotypes have the same values. ASRgenomics package has instructions to avoid an ill-conditioned matrix and calculate the G-inverse matrix to fit the GBLUP model in ASReml-R (Gezan et al. 2021).

#### Model 2: genomic-enabled prediction model on sparse phenotyping designs

The GP analysis was computed using the GBLUP model (Burgueño et al. 2012; Jarquín et al. 2014). The main effect of genotypes, the main effect of environments, the main impact of markers, and their interactions were modeled using random covariance structures that are functions of the genomic and environments covariates. GBLUP has been used to predict the not-tested lines across the environments on various sparse phenotyping designs; below is a summary of the prediction models.

7 $$Y_{ijk} = \mu \, + \, S_{i} + T_{j} + LT_{ij} + L^{\prime } G_{kn} + LTG_{ijk} + e_{ijkn}$$

Consider that Y_ijk_ represents the phenotypic response value of the k^th^ genotype/line in the i^th^ environments. The model includes a series of fixed and random effects that account for various sources of variation. The term µ denotes the overall mean, while S_i_ represents the fixed effect of the i^th^ environment. The random effects include T_j_, which captures the effect of the j^th^ tester, and captures the variation introduced by testers across genotypes and environments. Additionally, the interaction between the environment and the tester is modeled by LT_ij_ which describes how different testers respond across various environments. The term L’G_kn_ represents the interaction between the genomic effect of the k^th^ genotype or line and the tester, indicating how the genotypic differences influence the response under the effect of different testers. The higher-order interaction term, LTG_ijk_ captures the combined effects of environment, tester, and genotype, modeling more complex influences on the phenotypic outcome. This interaction term was assumed to follow a multivariate normal distribution with the following shape: gL ~ N(0; Z_g_GZ’_g_ Z_s_Z’_s_σ^2^LS) (Jarquín et al. 2014). The incidence matrices Z_g_ and Z_S_ were used to connect phenotypes to genotypes and environments. The variance component of the interaction is denoted by σ^2^LS, with the Hadamard product applied between the matrices Z_g_GZ’_g_ and Z_s_Z’_s_. The FA model was discovered as a more sparse strategy for fitting the complex covariance structure of a large number of environments (Burgueño et al. 2007, 2008, 2011, 2012; Crossa et al. 2004; Piepho 1998; Smith et al. 2001). The FA model for Cov (v_g,_v′_g_) is as follows: **(TT′+ £) ⊗ G.** where T is a J×K matrix of loading factors, the columns of T are associated with the environments of loadings for the m^th^ latent factor. Where £ was a J * J diagonal matrix with non-negative diagonal parameters i^th^ specific environmental genetic variances £v on the diagonal and zero covariance between environments, we consider that only one FA model contains two multiplicative elements. Thus, FA can be thought of as the linear regression of genotype and GP on environmental factors, with each genotype having its slope but sharing an intercept (Burgueño et al. 2007, 2008, 2011, 2012; Crossa et al. 2004; Piepho 1998; Smith et al. 2001). e_ijkn_∼N (0, R) is with R as the residual variance.

### Software

Mixed model equations have been used to calculate the adjusted values variance components and predict breeding values by the ASReml-R package in version R 4.1.2 (Butler et al. 2017). The G matrix was calculated by the AGHmatrix-R package (Amadeu et al. 2016). The ASRgenomics-R package calculated the G-inverse matrix (Gezan et al. 2021). RMs have been estimated by utilizing the STPGA package in R 4.1.2 software (Akdemir 2017). Allocation of lines into sets and evaluation of sparse phenotyping strategies were implemented using customized R 4.1.2 programs.

## Results

Five optimal-managed environments were used to evaluate all phenotypic traits. As established by the test crosses, the board sense heritability was used to quantify heritability across the five environments, including GEI. Heritability of GY and EPP was 0.75 and 0.64. The heritability of PH and EH was 0.93 and 0.91 in METs. PH and EH are highly heritable quantitative traits than EPP and GY.

### Sparse phenotyping approaches have pros and cons in terms of the AMs (Pearson correlation between the OBV vs PBV) and % of saved plots

**Fig. 3:**
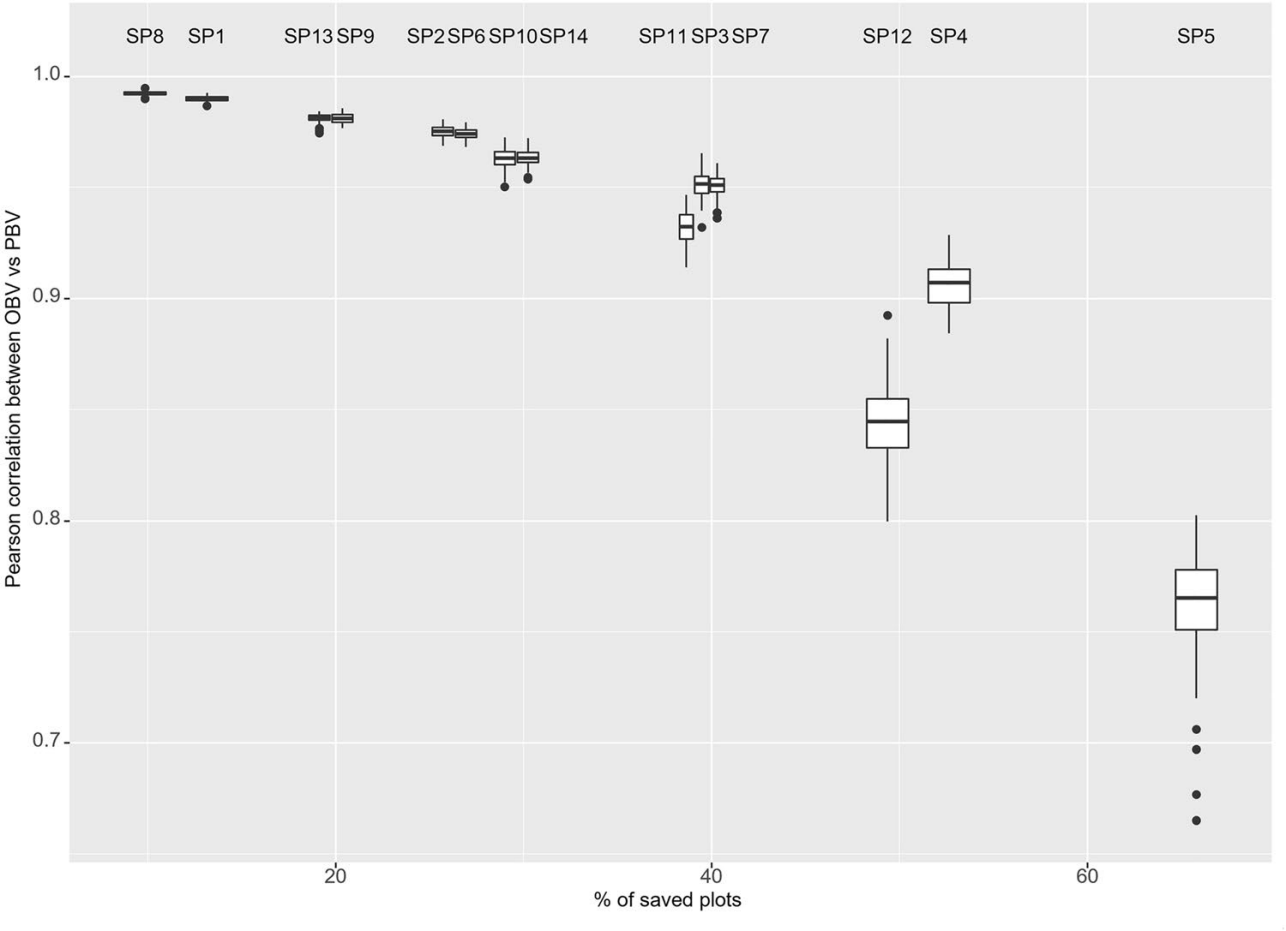
The figure has included the boxplots of the Pearson correlation between the observed and predicted lines of the grain yield genetic values by the % of the saved plots per sparse phenotyping design (SP1–SP14, *n* = 100 simulations per design)

The analysis has shown the performance of different sparse designs on the Pearson correlation from the 100 simulations and % of saved plots using METs. Breeders can make decision-based AMs (Fig. 3) to ensure how much the phenotyping can be reduced and the cost of precision using the different sparse phenotyping designs. The average Pearson correlation across 100 simulations ranged from 0.84 to 0.99 in GY (Fig. 3) by allocating 50% (154 lines) of the lines to the FS (SP8–SP14). In contrast, EPP and EH ranged from 0.75 to 0.99 (Suppl. Figs. 1 and 3). The correlations ranged from 0.81 to 0.99 between the SP8 and SP14 in PH (Suppl. Fig. 2). The designs of 30% (104 lines) of the lines allocated to the FS have shown a correlation from 0.76 to 0.99 (SP1–SP7) in GY (Fig. 3) and slightly similar for PH (0.72–0.99) (Suppl. Fig. 2) but changing the minimum in correlation for EH (0.64) and EPP (0.61) (Suppl. Figs. 1 and 3). The SP1–SP7 designs produced less accuracy than the methods that allocated more lines to the FS in four traits. The high phenotyping cost is reduced by assigning 30% of the lines to the FS from the SP1 to SP7 strategies in four traits.

The SP5 and SP12 sparse designs exhibited higher 65.78% and 49.35% of the saved phenotypic plots during our research to complete phenotyping designs. The SP5 and SP12 designs demonstrate a mean Pearson correlation of 0.76 and 0.84 in GY, 0.64 and 0.75 in EPP (Suppl. Fig. 3), 0.72 and 0.81 in PH (Suppl. Fig. 2), and 0.63 and 0.75 in EH (Suppl. Fig. 1). The number of not-tested environments changes from one to five environments per SS; the mean of Pearson correlation reduced slightly across the SP1–SP5 designs from 0.990 to 0.762 and the SP8–SP12 from 0.992 to 0.842 in GY while decreasing the phenotyping cost per plot across the designs (Fig. 3). The Pearson correlations reduced across the SP1–SP5 and SP8–SP12 ranged between the 0.63 and 0.99 and from 0.75 to 0.99 in EH and EPP (Suppl. Figs. 1 and 3) while PH ranged between the 0.72 to 0.99 and 0.75 to 0.99 (Suppl. Fig. 2). The SP4, SP5, SP11, and SP14 methods have shown more variations in Pearson correlation (Fig. 3) among the 100 simulations than other sparse phenotyping designs because of a smaller number of phenotyping plots tested (Suppl. Figs. 1, 2, and 3).

A high average Pearson correlation (Fig. 3) was achieved across the 100 simulations using a design in which only one environment was not tested for each SS, and 50% (154 lines) of the lines were allocated to the FS with a balanced design (SP8) in four traits (Suppl. Figs. 1, 2, and 3). About 9.86% of plots were saved by using the SP8 strategy. However, the mean Pearson correlation was 0.98 in EPP while 0.99 in GY, EH, and PH, indicating that SP8 is advantageous in terms of Pearson correlation. The balanced designs produced a higher accuracy Pearson correlation than the unbalanced designs. The % of saved phenotypic plots was increased using the unbalanced designs in four traits.

Suppl. Tables 2 and 3 show the average of the common % of the selected genotypes, mean of average rank, and differential of selection across the 100 simulations among the sparse designs and four phenotypic traits. The analysis (Suppl. Tables 2 and 3) has shown that AMs are minimized and maximized according to the sparse methods and traits. The Pearson correlations between the OBV and PBV for GEI were calculated, and the results Suppl. Fig. 5 illustrates the minimum and maximum Pearson correlations ranging from 0.97 to 1 across the sparse phenotyping designs in GY. Suppl. Fig. 4 shows the box plots of the Pearson correlations between the OBV and PBV for not-tested SSs for the environment according to designs.

### Positive Pearson correlation between the AMs and RMs can be helpful to optimize the line allocation

**Fig. 4:**
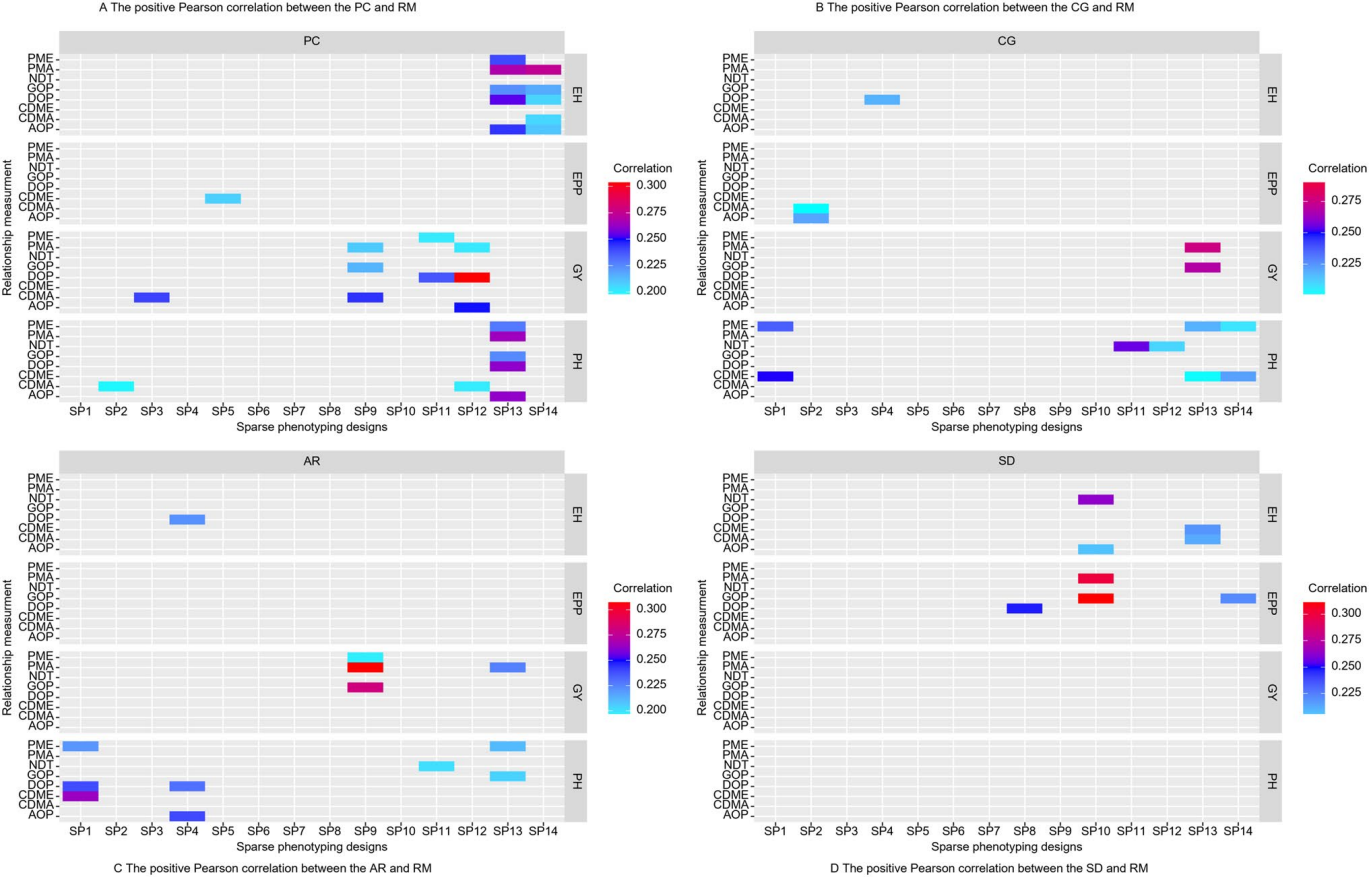
Pearson correlations between RMs (PME; PEVMEAN, PMA; PEVMAX, NDT; neg dist in a train, GOP; GOPTPEV, DOP; DOPT, CDME; CDMEAN, CDMA; CDMAX, AOP; AOPT) and AMs (PC: Pearson correlation between OBV and PBV; CG: Common proportion of selected genotypes; AR: Average rank of selected genotypes; and SD: Differential of selection) by trait and sparse design. Only significant correlations (*p* < 0.05) are colored

A positive correlation indicates that the RMs are related to the AMs on sparse phenotyping designs; therefore, maximizing and minimizing the relationship measurement produces better accuracy and can be used to optimize line allocation. Figure 4 illustrates the significant Pearson correlations between the four AMs and eight different RMs by the various sparse designs among the quantitative traits.

#### A positive significant Pearson correlation between the RMs and Pearson correlations between OBV and PBV (PCs) can be helpful

The analysis (Fig. 4A) has illustrated that there is indeed a significant positive correlation coefficient between the RMs and PCs to enhance the PCs accuracy, which can help optimize the line allocation utilizing sparse phenotyping experiments. The PCs and RMs have not displayed significant association with SP1, SP4, SP6, SP7, SP8, and SP10 designs and the four traits (EH, EPP, PH, and GY). By using the SP5 among all sparse methods in the EPP, the CDME has demonstrated a significant Pearson correlation of 0.21. The minimum correlation is 0.20 using the CDMA on the SP2 and SP12 in the PH. The Pearson correlation ranged from 0.23 and 0.26 by utilizing the GOP, PME and PMA, DOP, and AOP measurements on SP13 in the PH. Exploring the GOP, PME, PMA, DOP, and AOP in the PH, the SP13 design had correlations between 0.23 and 0.26. The correlation ranged from 0.20 to 0.25 in SP3 (CDMA), SP9 (CDMA, GOP, and PMA), SP11 (DOP and PME), and SP12 (PMA and AOP) in the GY. The SP13 (AOP, DOP, GOP, PMA, and PME) and SP14 (AOP, CDMA, DOP, GOP, and PMA) significantly correlated from 0.21 to 0.27 in the EH. The maximum significant Pearson correlation is produced at 0.30 using the DOP and SP12 design in the GY among all methods and traits. The second highest correlation is 0.28 using the SP3 and PMA in the GY.

#### Few measurements show a positive Pearson correlation between the RMs and CG

Above (Fig. 4B) displayed that a positive significant Pearson correlation of the RMs on the CG improves the common % of selected lines by optimizing the line allocation in sparse phenotyping designs. The SP3, SP5, SP6, SP7, SP8, SP9, and SP10 designs have shown a significant Pearson correlation between the RM and CG across the four traits. A positive significant Pearson correlation occurred in the DOP in EH with 0.22 on the SP4. The SP2 can accomplish the minimum 0.20 and 0.22 correlations by employing the AOP and CDMA in the EPP. The correlations for SP1, SP11, and SP14 utilizing the PME, NDT, and CDME in the PH ranged from 0.20 to 0.26. The highest Pearson correlation was obtained at 0.27 and 0.28 on SP13 using PMA and GOP in the GY.

#### Very few measurements showed a significant correlation between AR and RMs among the RMs, designs, and traits

The result (Fig. 4C) illustrates a positive Pearson correlation between the AR and RM in sparse phenotyping designs, which can help optimize the line allocation using AR. The EPP has not observed significant correlations between the RM and AR in sparse methods. Only the SP4 produced the 0.22 significant positive correlation with the assistance of the DOP in EH. The minimum 0.20 correlation resulted in SP9 using the PME in GY, SP11 using the NDT, and SP13 using GOP in PH. The significant correlations ranged from 0.21 to 0.26 on the SP1 (CDME, DOP, and PME), SP4 (AOP and DOP), and SP13 (PME) in the PH. The highest correlation of 0.31 between PMA and AR was observed for GY under the SP9 design.

#### GY, PH traits, and SP1–SP7 designs do not show a positive significant Pearson correlation between the RMs and SD

Figure 4D represents how well the positive correlations between the RM and SD work in sparse phenotyping designs that use METs to optimize the line allocation. The SD and RM have not shown a significant Pearson correlation on sparse phenotyping methods in GY and PH. The SP8 and SP14 designs have shown a significant correlation of 0.22 and 0.25 to using the GOP and DOP in the EPP. Only the AOPT, NDT on SP10 and the CDMA, and CDME on SP13 have significant correlations between 0.21 and 0.26 in the EH. The PMA and GOP were used on the SP10 in EPP, resulting in high significant correlations between 0.30 and 0.31.

Our research used several sparse phenotyping designs, and it is uncertain which RMs, AMs, and sparse phenotyping provide the most precise significance to optimize the line allocation. The results (Fig. 4) have demonstrated low (0.20–0.31) significant Pearson correlations between the four AMs and the efficiency of the RMs on sparse phenotyping methods among the four traits. The significant and not significant Pearson correlations have depended upon the relationship between the FS and SS, sparse phenotyping designs, the structure of the data, and traits. The results show that positive significant Pearson correlations between the AM and RM were utilized to enhance the accuracy of measurements to optimize the line allocation in sparse testing. The findings of sparse phenotyping designs imply that pre-selection can be achievable from the positive correlation. The low significant and negative (<0.05) Pearson correlation between RMs and four AMs demonstrate that RMs cannot be obtained to optimize line allocation in sparse phenotyping design to reduce the phenotypic resources without compromising the precision.

## Discussion

Phenotypic is expensive to conduct a large number of breeding trials including line by tester in METs. These expenses impose limitations on the number of plots to which genotypes can be given to the FS and SS. While the price of genotyping is decreasing and should continue to do so over the future decades, the cost of phenotyping seems expected to stay high. The present study mainly focused on measuring the relationship between the full and sparse genomic sets using eight RMs on sparse phenotyping designs to determine which RMs help optimize the line allocation in METs to improve the precision and reduce the cost of the phenotyping compared with the complete phenotyping field trials.

### Importance of different sparse phenotyping designs in METs

Due to limited economic resources per plot unit, the plant breeding programs must plant a small fraction of plots while utilizing molecular and field observation resources to improve genetic gains. Given fixed costs, breeders should assess how many lines can be genotyped and how many can be evaluated in the field to maximize genetic benefits (Atanda et al. 2021a, b; Crespo-Herrera et al. 2021; Jarquin et al. 2020). According to an earlier study, sparse phenotyping designs with GP in METs can minimize costs and boost genetic gain (Atanda et al. 2021a, b; Jarquin et al. 2020). The results have shown that sparse phenotyping reduces the phenotyping cost and maximizes precision. In this case, we can increase the population size and number of phenotyped environments to improve genetic gain. The results are similar to the previous research (Atanda et al. 2022; Atanda et al. 2021a, b; Crespo-Herrera et al. 2021; Jarquin et al. 2020).

### Application of GP in sparse phenotyping designs and performance of sparse phenotyping methods in METs among the prediction accuracy

This study explored and compared the complete phenotyping field trials with the sparse phenotyping approaches using GP to enhance prediction accuracy throughout the METs with genotype advanced based on the GBLUP with FA model by reducing phenotyping cost. Sparse testing uses GP application to predict untested lines in different but genetically associated environments by utilizing information from closely related individuals within and across environments (Atanda et al. 2022; Atanda et al. 2021a, b; Atanda et al. 2021a, b; Burgueño et al. 2012). According to Crossa et al. (2017) review, the sparse phenotyping is indeed a cross-validation 2 (CV2) strategy because some lines were observed in some environments but not in others, utilizing the GP to increase genetic gain and testing environments (Burgueño et al. 2012).

Montesinos-Lopez et al. (2022) implemented the incomplete block design (IBD) on sparse phenotyping designs to obtain efficient prediction accuracy, and they found that allocation of the lines IBD is time-consuming and concluded that IBD methods enhance the prediction in sparse phenotyping designs. Recently, Montesinos-López et al. (2023) researched by exploring the four sparse designs on large and small datasets with single-trial and multi-trial analysis and found that the large datasets and multi-trials are helpful in enhancing the prediction accuracy. However, our research designed 14 sparse designs, estimated the AMs and applied RMs to identify whether RMs help mimic the sparse phenotyping designs. Our study found the high AMs on different sparse methods significantly useful to reduce the resource allocation in METs in maize.

Atanda et al. (2021a, b) observed that borrowing information across environments enhanced the prediction by using sparse testing compared to test half and predict half. An approach that optimizes genotype coverage across environments should improve predictability. The FA is a compressed model for fitting a large number of environments in METs. It helps use latent factors that generate correlations between existing environments, and the FA is a specific structure for that matrix of correlation to increase the 6% prediction accuracy (Burgueño et al. 2011, 2012). In our case, we used FA to model a variance–covariance matrix (genetic variance–covariance between environments) because the structure of the matrix is flexible and the method parsimonious; few numbers of parameters can model a complex matrix to enhance the prediction accuracy in METs.

Atanda et al. (2022), Crespo-Herrera et al. (2021), and Jarquin et al. (2020) studied overlap and non-overlapped genotypes in sparse designs to maximize genetic gain, increase selection intensity, and save resources. The overlapping test is one set of lines in all environments, and the non-overlapping criteria evaluate the multiple sets of lines within environments (Jarquin et al. 2020). Atanda et al. (2022), Crespo-Herrera et al. (2021), and Jarquin et al. (2020) found that sparse testing based on GP could efficiently increase the number of testing environments while also establishing the intensity of selection in the early yield testing phase without raising the breeding expenditure. Our results showed that using the different sparse designs offers advantages and disadvantages in terms of AMs and % of saved plots. Utilizing the GP and sparse strategies maximizes the accuracy and reduces field evaluation resources to improve the genetic gain. We have not employed overlap or non-overlap sparse methods in our research. Still, our results are similar to Jarquin et al. (2020) results in terms of the prediction accuracy and differential of the selection by using different sparse testing methods. During our research, the selection of the lines among the FS and SSs per sparse methods determined the selection differential. Akdemir et al. (2015) and Jarquin et al. (2020) have shown that the prediction accuracy changed dramatically by reducing the number of lines in the FS. We found similar results by assigning the two different % of lines to the FS; we could see an evident variation in prediction accuracies between the 50 and 30% of lines allocated to the FS designs. Maximizing the AMs mainly depended upon sparse phenotyping designs, primarily % of lines allocated to the FS, the number of environments phenotyped for each SS, and balanced sparse designs.

The relationship between heritability and GP accuracy is a crucial aspect of quantitative genetics. Several studies highlight the significant impact of varying heritability levels on the accuracy of GP models. For instance, Akdemir and Sánchez (2016) demonstrated that higher heritability leads to improved prediction accuracy in simulated datasets, while lower heritability diminishes it. Additionally, Habier et al. (2007) found that high heritability markedly increases the precision of marker effect estimates, whereas low heritability reduces accuracy. Isidro et al. (2015) reviewed various optimization approaches in GP, asserting that environments with higher heritability and proper training set optimization significantly enhance prediction accuracy. Collectively, these studies underscore the critical influence of heritability on genomic prediction, reinforcing the validity of our findings that utilize raw phenotypic data for estimating heritability in the context of our research. These findings suggest that PH and EH are more heritable traits compared to EPP and GY, aligning with the literature that emphasizes the importance of heritability in enhancing prediction performance. The effectiveness of integrating heritability estimates with optimized phenotyping strategies to improve the accuracy of genomic predictions.

### RMs on sparse phenotyping design compared with the four AMs

Isidro y Sánchez and Akdemir (2021) reviewed training population optimization in plants and the major issues connected with GP optimization, including population size, the relationship between training and testing sets, updating of the training set, and the use of multiple packages and techniques for the training set implementation in GP. Our research included optimization criteria for calculating the genomic relationship between the FS and SSs with the help of different RMs to mimic the sparse testing. The size and structure of the FS sample are crucial performance criteria for GP algorithms. Optimizing the training set by selecting the most predictive individuals instead of utilizing a random sample increases predicting capacity (Akdemir et al. 2015; Rincent et al. 2012).

Our research could not include optimizing the FS by using the RMs but utilized the RMs to calculate the genomic relationship between the FS and SS on sparse phenotyping designs. The results clearly show the positive and negative Pearson correlation between the RMs on AMs. The positive Pearson correlations demonstrate that the RMs can help obtain and enhance the prediction accuracy and optimize the METs in sparse phenotyping designs. The prediction AMs showed better positive results on the RMs than the three measurements. In this study, Windhausen et al. (2012) focused on the significance of relatives for the composition of the training set and how to update the training set to enhance GP across generations. They highlighted how the training set should be composed in terms of similarity between the training and testing sets. However, they did not execute an optimization procedure; the training set was chosen randomly. A random sample of genotypes from a dataset is risky because it may result in inadequate coverage of the overall genetic space, particularly when the complete dataset has a population structure (Isidro et al. 2015; Windhausen et al. 2012). They highlighted how the training set should be composed with the relationship between the training and testing sets. However, they could not execute an optimization procedure. The training set was chosen randomly and considered how helpful training set optimization is in the breeding program. The original papers for CD mean by Rincent et al. (2012), STPGA from Akdemir et al. (2015), and stratified sampling by Isidro et al. (2015) describe a situation where genotypes are available for an extensive collection of germplasm. However, still, there are not enough resources to phenotype with less cost. In this case, an optimization algorithm determines an optimal training set. Those researchers generated the results of the RMs utilized to optimize the line allocation, but they have not researched training and testing sets in sparse phenotyping schemes.

The phenotype heritabilities and the choice of the training population in relation to the test population are two factors that could affect how well GP performs (Crossa et al. 2017). Optimizing the reliability of these contrasts instead of the predictions per selection needs to consider the differences between the individuals that make up the target population. This helps to keep closely related individuals from being selected for training population formation (Rincent et al. 2012). Isidro et al. (2015) have demonstrated that optimizing a population’s training depends on the population and the trait. Akdemir et al. (2015), Isidro et al. (2015), and Rincent et al. (2012) used a principal component-based approach that made computing more efficient and chose training populations based on how they related to a certain target population rather than how they related to each other within the training population. Atanda et al. (2021a, b) and Lopez-Cruz et al. (2015) have focused on how the training size and the degree of similarity between the training set and the testing set affect the ability to predict.

The AMs are significantly affected by RMs. The AMs are mainly affecting the heritability of the traits. The high heritable trait produced high accuracy in GP. More consideration should be given to historical information from METs research performed over several years. With such knowledge, GP performance seems more likely to improve, accelerating breeding results with significantly increased genetic gain. Habier et al. (2010) pointed out that the accuracy of GP depends significantly on the relationship between training and testing populations. Several research authors showed that when unrelated lines are incorporated into the training set, the accuracy of the predictions reduces. Marker density and population size significantly affect how well a prediction can be initiated. The % of the genotypes that were pre-selected for genetic improvement. The smaller the number, the bigger the selection differential.

During our research, eight RMs were used on AMs to determine how well RMs useful in optimizing the line allocation in sparse phenotyping experiments. The RMs show the advantages and disadvantages of optimizing the line allocation in sparse phenotyping schemes. In sparse phenotyping, designs have resulted in low significant correlations between the relationships by AMs. Still, a few RMs have shown (Fig. 4) a significant Pearson correlation on sparse phenotyping designs. The various factors may affect the results: (1) High heritability, therefore, it is difficult to improve accuracy because it was already relatively high; (2) AMs calculated across all lines (FS and SSs), not only the sparse phenotyped lines; (3) high genetic correlation between environments; (4) a population with low genetic variability; (5) the comparison of the predicted value with the GBLUP of the complete dataset, usually the literature compare predicted values with the simple means of the genotypes, in the best of the cases, the adjusted means (BLUE), i.e., an observed value including environmental variability; (6) RMs utilized in sparse phenotyping designs; (7) only 1 year and five environments; (8) each line has evaluated in at least one or two environments; and (9) the lines phenotyped across all environments.

The above analysis, the factors optimization of line allocation using RMs, can be possible from the positive Pearson correlation between RMs and AMs in sparse phenotyping designs. This challenge, for which we have a collection of candidates and evaluate them in sparse methods by using genetic data to predict the missing values and then have the order mean of the lines, is not accessible to measurements. We already have line information because the majority of lines have been tested in the field. The positive Pearson correlation between the RMs and AMs demonstrated that the breeders can use RMs to determine the optimal training set using sparse phenotyping methods. Breeders can operate directly on the training set by selecting the sparse phenotyping design and randomly distributing the lines. It would help the breeders to enhance the accuracy of measurements and reduce the phenotyping cost by utilizing the sparse phenotyping strategies in METs.

## Conclusion

In general, RMs between training and testing populations have optimized training populations using complete phenotyping designs. During our study, the positive correlations between the AMs and RMs have shown that RMs are helpful in optimizing line allocation in sparse phenotyping design. The negative correlations between the AMs and RMs have not been helpful in optimizing the sparse designs in METs. The negative correlations between the AMs and RMs occurred because of the specific characteristics of the study which can be a high heritability of the traits, a medium to high genetic correlation between environments, low genetic variability between lines, and the fact that all the lines are phenotyped at the end explaining the opposite results with the literature results. According to our results, in similar situations, genotyping in advance is not a bottleneck for plant breeding since the genotyping can be performed late in the growing cycle to have genotype breeding value on time for the next cycle. Further research in more critical conditions could provide a better understanding of the situations in which optimizing the training and testing set and line allocation using sparse phenotyping data could be helpful in plant breeding. It is advantageous for breeds to execute the optimizations approach for pre-selection utilizing RMs on sparse phenotyping models before conducting field trials.

## Supplementary Information

Below is the link to the electronic supplementary material.

Supplementary file1 (PDF 399 kb)

## Data Availability

The datasets generated during and/or analyzed during the current study are available in the CIMMYT DATAVERSE repository, data.cimmyt.org.

## Abbreviations

AMs: Accuracy measurements
AR: Average rank of selected genotypes
BLUEs: Best linear unbiased estimators
CD: Coefficient of determination
CG: Common proportion of selected genotypes
CIMMYT: International Maize and Wheat Improvement Center
EH: Ear height
EPP: Ears per plant
FA: Factor analytic
FS: Full set
GBLUP: Genomic best linear unbiased predictor
GEI: Genotypic by environmental
GY: Grain yield
GMP: Global maize program
GP: Genomic prediction
IBD: Incomplete block design
METs: Multi-environmental trials
OBV: Observed breeding value
PBV: Predicted breeding value
PC: Pearson correlation between OBV and PBV
PEV: Prediction error variance
PH: Plant height
rAmpSeq: Repeat amplification sequencing
RMs: Relationship measurements
SD: Differential of selection
STPGA: Selection of training populations by genetic algorithm
SP-1: Sparse phenotyping-1
SS: Sparse set
SSs: Sparse sets
