# Supplementary material for: Optimization of sparse phenotyping strategy in multi-environmental trials in maize
Source: Theor Appl Genet. 2025 Feb 28;138(3):62. doi: 10.1007/s00122-025-04825-y (PMC11868319; doi:10.1007/s00122-025-04825-y)
Supplement: Supplementary file 1 — Supplementary file1 (PDF 399 kb) [file 122_2025_4825_MOESM1_ESM.pdf]

Supplementary Information (SI)

Optimization of Sparse Phenotyping Strategy in Multi-Environmental Trials in Maize

Authors and Affiliations

Srinivasa Reddy, M.[1 4], Beyene, Y.[2], Gültas, M.[3], **Burgueño*, J,[4]** and S. Griebel[5].

* Corresponding author s.mothukuri@uq.edu.au, J.Burgueno@cgiar.org

[1] Faculty of Agriculture, University of Göttingen, Büsgenweg 5, 37077 Göttingen, Germany. 0000-0003-2232-6100.

[2] International Maize and Wheat Improvement Center, Global Maize Program. ICRAF House, United Nations Avenue, Gigiri, Nairobi, Kenya. 41Village Market-00621. 0000-0001-7221-26173.

[3] Faculty of Agriculture, South Westphalia University of Applied Sciences, Lübecker Ring 2, 59494, Soest, Germany. 0000-0003-3297-3192.

[4] International Maize and Wheat Improvement Center, Biometrics and Statistics Unit. Carretera México-Veracruz, Km. 45, El Batán, Texcoco, CP 56237, México. 0000-0002-1468-4867.

[5] Georg-August-University Göttingen, Faculty of Agricultural Sciences, Department of Crop Sciences, 37075 Göttingen, Germany. 0000-0001-6931-5711.

2.0 Material and Methods

2.1 Data sets

Supplementary Table 1: Field phenotyping design in one environment. 912 test crosses were divided into 7 sets; each set was tested in an alpha (0,1) experimental design. The experimental field design is similar in every environment; each environment is described in the table.

Set	Number of Lines	Number of TC	Number of Checks	Number of Blocks	Block size (Plots)	Number of Plots
1	45	135	5	28	5	140
2	61	183	5	47	4	188
3	46	138	5	34	4	152
4	26	78	6	14	6	84
5	50	150	5	31	5	155
6	38	114	6	20	6	120
7	38	114	6	20	6	120

3.1 Sparse phenotyping approaches have pros and cons in terms of prediction accuracy and saved plots.

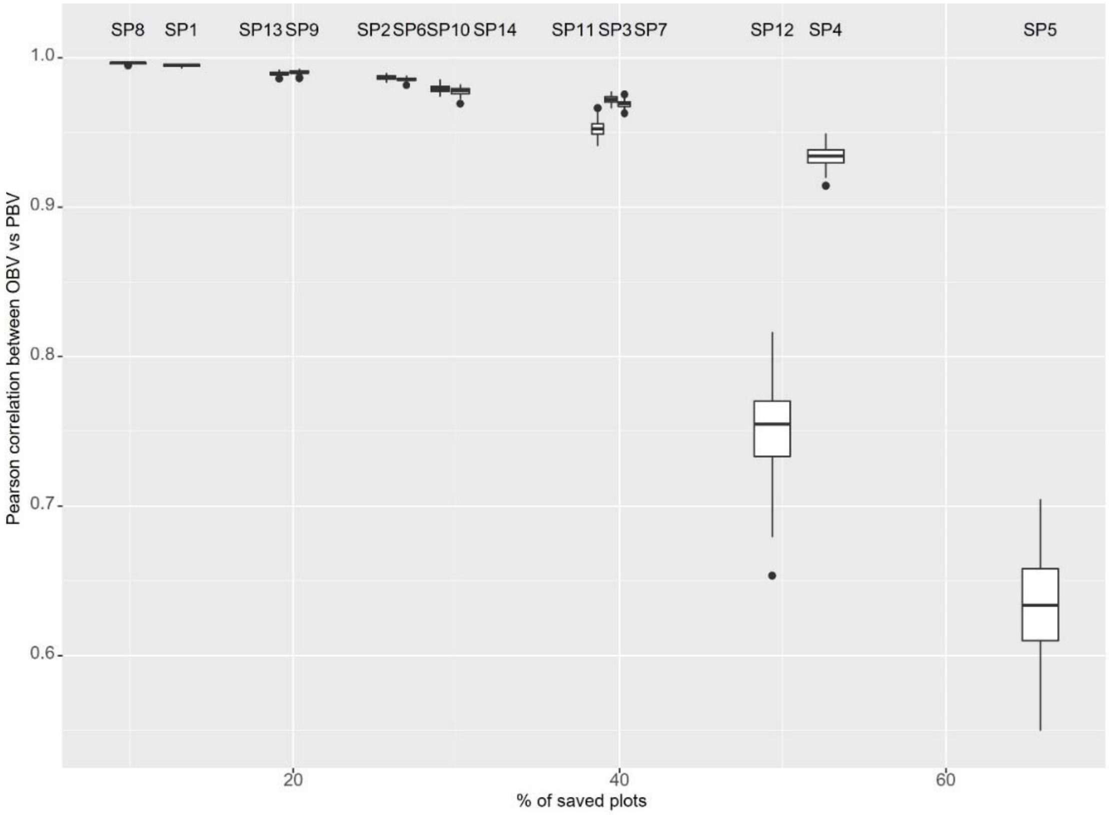

Supplementary Figure 1: The figure illustrates the boxplot of the Pearson correlation between the observed and predicted lines of the EH genetic values by the percentage of the saved plots per sparse phenotyping design (SP1 – SP14, n = 100 simulations per design).

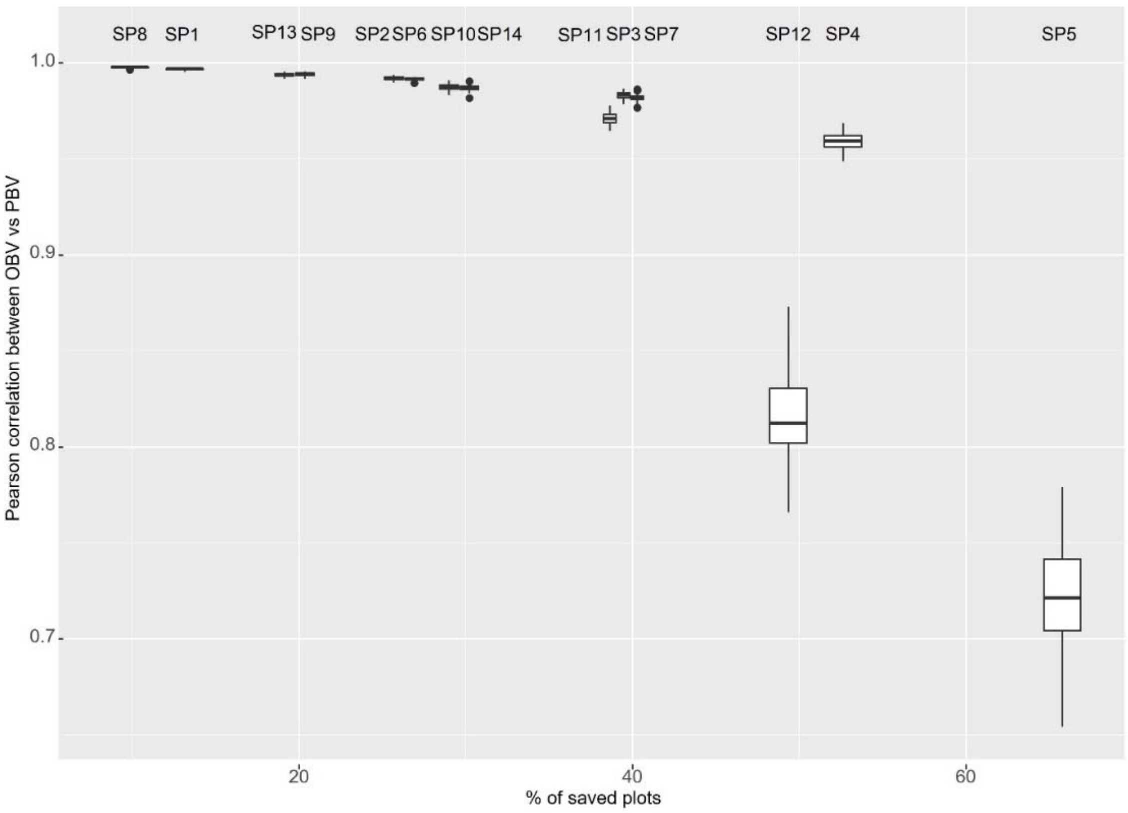

Supplementary Figure 2: illustrates the boxplot of the Pearson correlation between the observed and predicted lines of the PH genetic values by the percentage of the saved plots per sparse phenotyping design (SP1 – SP14, n = 100 simulations per design).

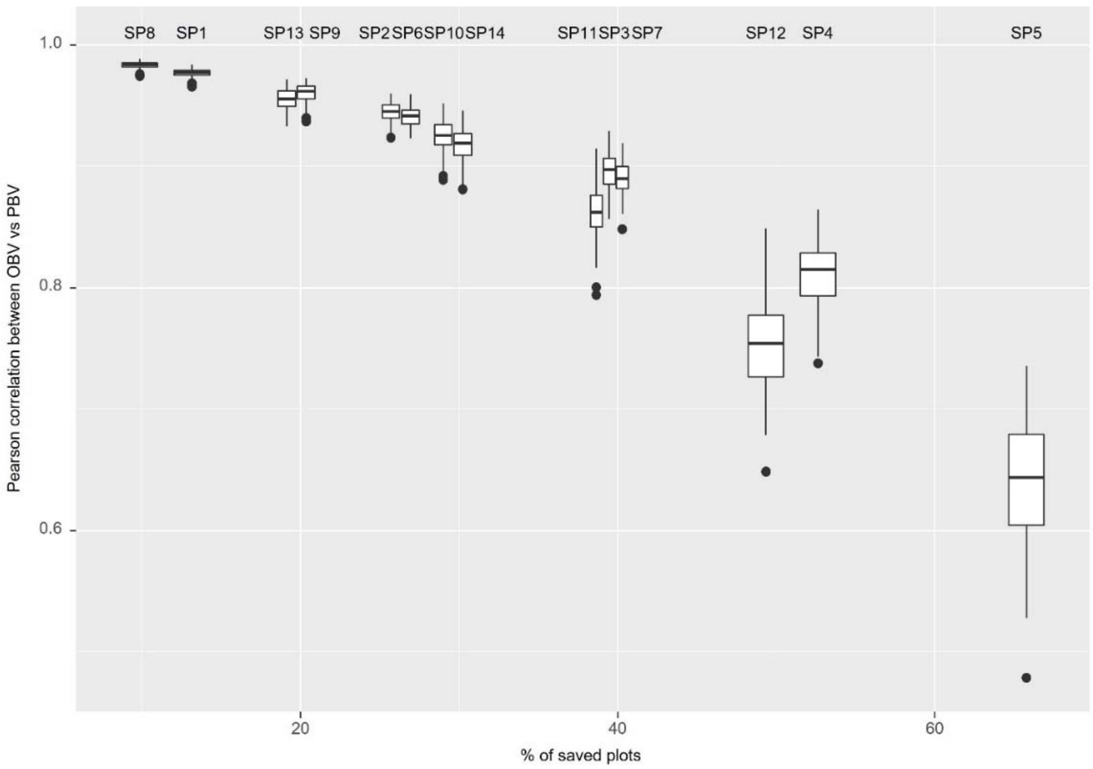

Supplementary Figure 3: The figure shows the boxplot of the Pearson correlation between the observed and predicted lines of the EPP genetic values by the percentage of the saved plots per sparse phenotyping design (SP1 – SP14, n = 100 simulations per design).

2.2. The high PC between the not-tested sparse sets per MET according to sparse phenotyping designs.

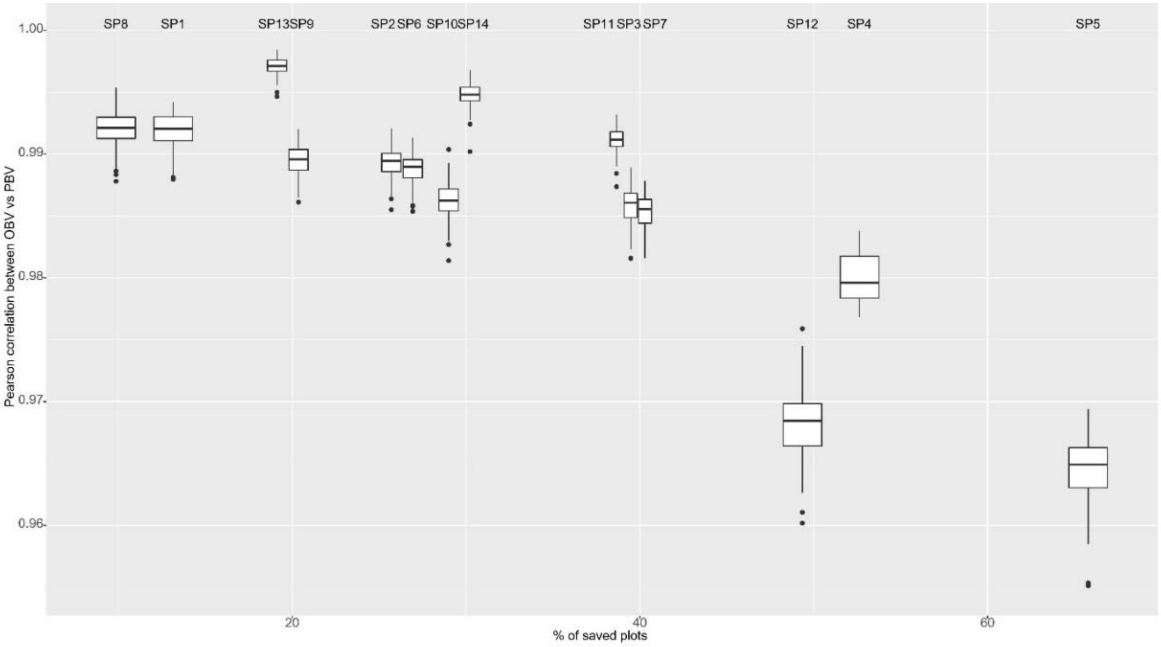

Supplementary Figure 4: Above the figure shows the boxplot of the Pearson correlation between the observed and predicted lines of not tested sparse set per environment among the sparse phenotyping designs and the GY genetic values by the percentage of the saved plots per sparse phenotyping design (SP1 – SP14, n = 100 simulations per design).

2.3. The high Pearson correlation between the OBV vs PBV was obtained from the GEI for the GY

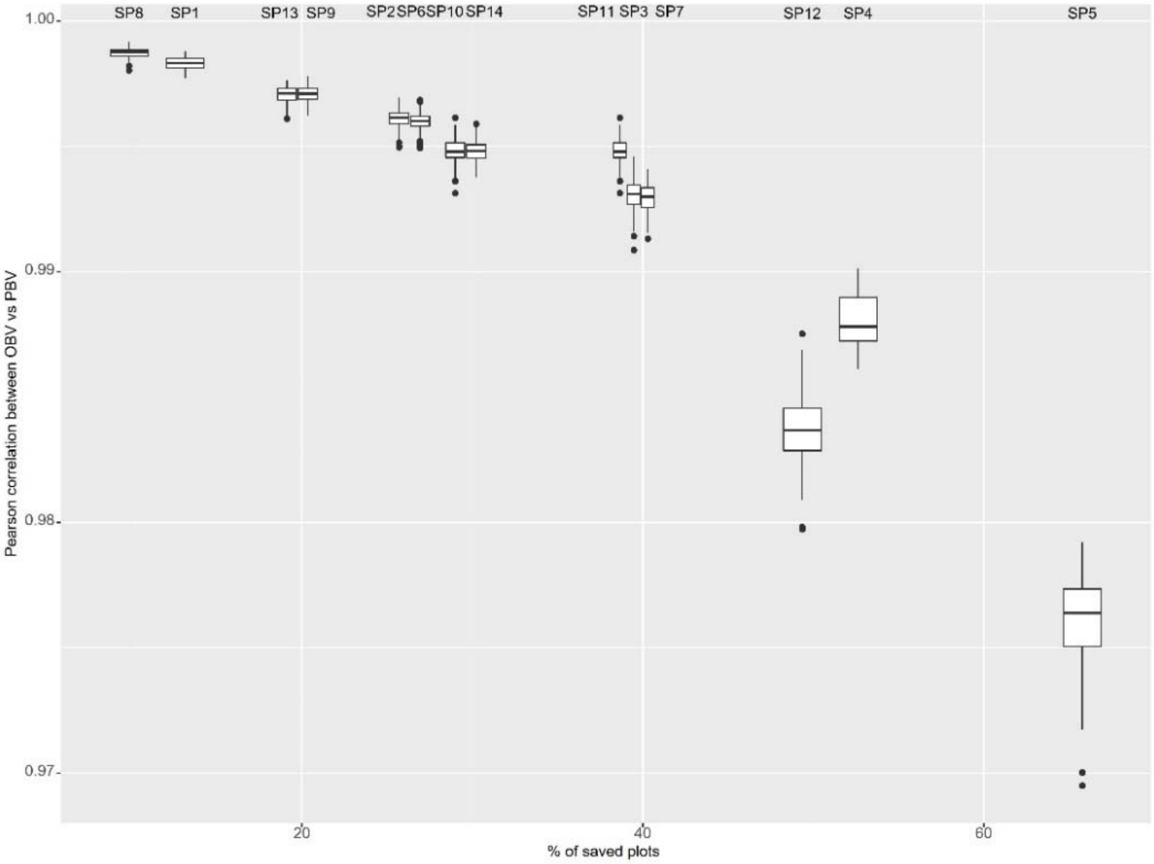

Supplementary Figure 5: The figure illustrates the boxplot of the Pearson correlation between the observed and predicted GEI of the GY genetic values by the percentage of the saved plots per sparse phenotyping design (SP1 – SP14, n = 100 simulations per design).

3.2 Average accuracy measurements across the 100 simulations for different sparse phenotyping designs

	Average of the Pearson correlations				Average of the common % of selected lines			
**Sparse Design**	**Grain Yield**	**Ear Per Plant**	**Plant Height**	**Ear Height**	**Grain Yield**	**Ear Per Plant**	**Plant Height**	**Ear Height**
SP1	0.99	0.97	0.99	0.99	91.9	88.96	95.4	89.2
SP2	0.97	0.94	0.99	0.98	86.6	82.16	93.1	85.1
SP3	0.95	0.89	0.98	0.97	81.2	75.3	87.4	81.7
SP4	0.9	0.81	0.95	0.93	72.6	66.26	79.9	75
SP5	0.76	0.64	0.72	0.63	55.36	49.73	51.1	48.6
SP6	0.97	0.94	0.99	0.98	87.1	82	91.8	85.7
SP7	0.95	0.89	0.98	0.96	80.63	75.66	86.6	81.2
SP8	0.99	0.98	0.99	0.99	93.13	90.86	96.5	91
SP9	0.98	0.96	0.99	0.99	88.3	86.66	93.1	88.9
SP10	0.96	0.92	0.98	0.97	84.1	79.96	89.2	83.7
SP11	0.93	0.86	0.97	0.95	77.9	72.43	83.7	80.7
SP12	0.84	0.75	0.81	0.75	65.3	60.23	62.5	60.3
SP13	0.98	0.95	0.99	0.98	89.1	85.36	93.1	88.9
SP14	0.96	0.91	0.98	0.97	84.8	79.93	89.2	85.2

Supplementary Table 2: The Average Pearson correlation and % of commonly selected lines for each sparse phenotyping design and trait. The top 30 lines from 304 lines in the OBV and PBV. The averages were calculated by using the 100 simulations in each sparse phenotyping design.

	Mean of the average rank.				Average of the differential of selection			
**Sparse Design**	**Grain Yield**	**Ear Per Plant**	**Plant Height**	**Ear Height**	**Grain Yield**	**Ear Per Plant**	**Plant Height**	**Ear Height**
SP1	16.15	16.74	15.78	16.96	1.05	0.055	16.14	10.92
SP2	17.57	18.9	16.24	17.97	1.03	0.053	16.06	10.84
SP3	19.89	22.15	17.76	19.72	1	0.05	15.8	10.67
SP4	24.57	29.88	20.24	23.85	0.95	0.046	15.38	10.29
SP5	39.64	52.53	44.01	53.53	0.81	0.035	12.04	7.62
SP6	17.3	18.79	16.51	18.1	1.03	0.053	16.01	10.83
SP7	19.77	22.04	17.89	20.11	1	0.051	15.79	10.64
SP8	16.01	16.39	15.69	16.7	1.05	0.055	16.16	10.94
SP9	17.25	17.6	16.19	17.26	1.03	0.054	16.07	10.89
SP10	18.63	20.09	17.26	19.08	1.02	0.052	15.89	10.73
SP11	22.16	25.83	19.12	21.44	0.98	0.048	15.57	10.49
SP12	31.1	38.76	32.45	37.96	0.89	0.042	13.57	8.9
SP13	16.93	18.03	16.34	17.35	1.04	0.053	16.04	10.89
SP14	18.8	20.4	17.29	18.9	1.02	0.052	15.88	10.75

Supplementary Table 3: The Mean of the average rank and differential of selection lines for each sparse phenotyping design and trait. The top 30 lines from 304 lines in the OBV and PBV. The averages were calculated by using the 100 simulations in each sparse phenotyping design.

3.3 A Positive Pearson correlation between the accuracy measurements and relationship measurements can be useful to optimize the line allocation

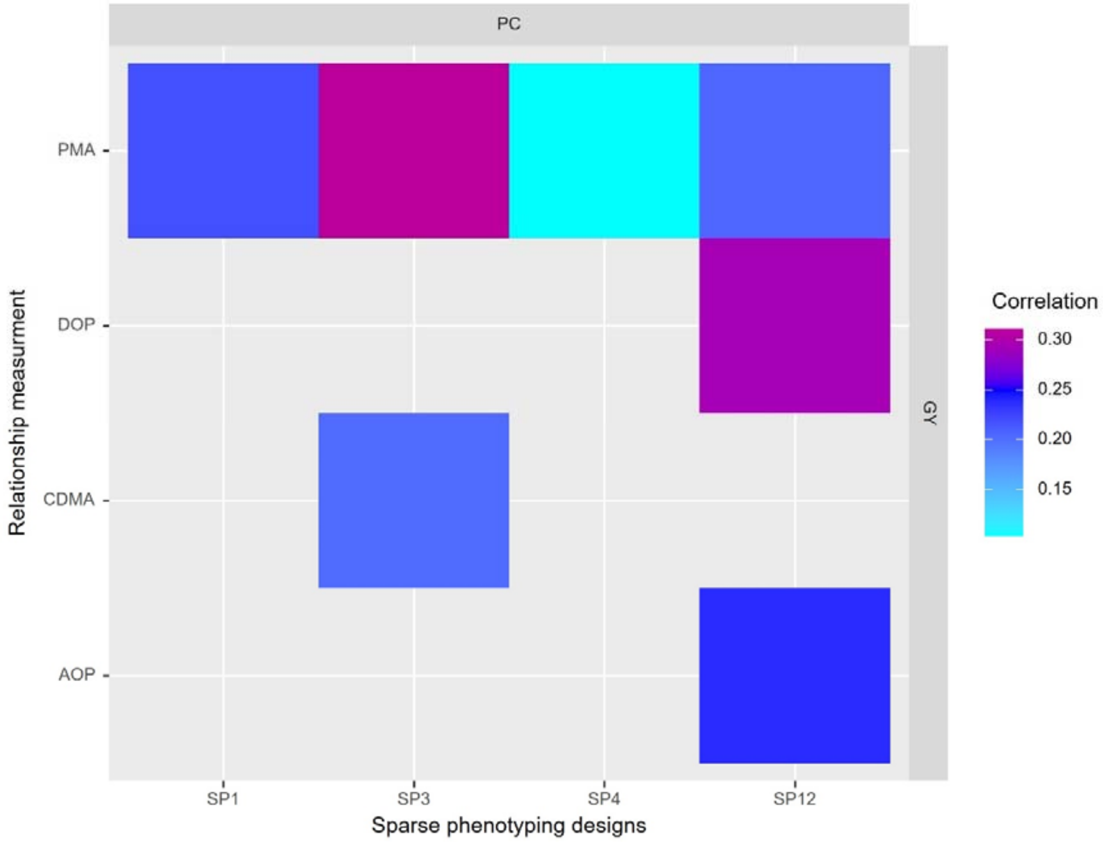

Supplementary Figure 6: Pearson correlations between relationship measurements (PMA; PEVMAX, DOP; DOPT, CDME; CDMA; CDMAX, AOP; AOPT) and accuracy measurements (PC: Pearson correlation between OBV and PBV for not tested sparse sets) by GY and sparse design. Only significant correlations ($p<0.05$) are colored.

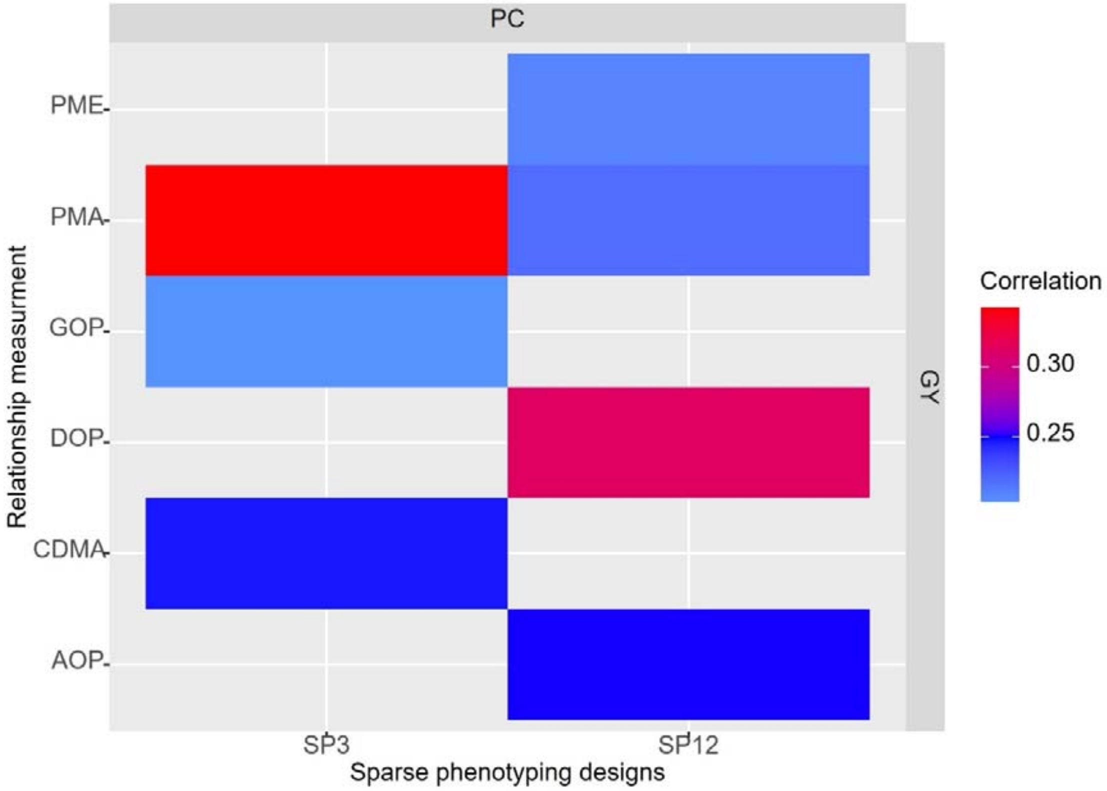

Supplementary Figure 7: Pearson correlations between relationship measurements and accuracy measurements (PC: Pearson correlation between OBV and PBV for line by environmental predictions) by GY and sparse design. Only significant correlations ($p<0.05$) are colored.
